# Real-world clinicopathological features and outcome of thymic neuroendocrine tumors: a retrospective single-institution analysis

**DOI:** 10.1186/s13023-022-02366-x

**Published:** 2022-06-06

**Authors:** Yeye Chen, Jiaqi Zhang, Mengxin Zhou, Chao Guo, Shanqing Li

**Affiliations:** Department of Thoracic Surgery, Peking Union Medical College Hospital, Chinese Academy of Medical Sciences and Peking Union Medical College, No. 1, Shuaifuyuan, Dongcheng District, Beijing, 100730 China

**Keywords:** Thymic neuroendocrine tumors, Clinicopathological features, Prognosis

## Abstract

**Background:**

Thymic neuroendocrine tumors comprise a heterogeneous group of rare diseases. This study aimed to investigate the real-world clinicopathological features and treatment outcomes of thymic neuroendocrine tumors.

**Results:**

A total of 104 patients diagnosed with thymic neuroendocrine tumors in a single institution from 1983 to 2021 were eligible. Fourteen (13.46%) and 28 (26.92%) patients diagnosed with thymic neuroendocrine tumors suffered from multiple endocrine neoplasia and ectopic adrenocorticotropic hormone syndrome, respectively. Ninety-seven (93.27%) patients underwent surgical resection, including 79 (81.44%) with radical resection. Except for 5 patients lost during follow-up, the 1-, 3- and 5-year overall survival rates were 91.8%, 70.2% and 54.6%, respectively. The median overall survival was 61.57 months. Multivariate analysis revealed that years at diagnosis (HR 0.559, 95% CI 0.364–0.857, *p* = 0.008), radical resection (HR 2.860, 95% CI 1.392–5.878, *p* = 0.004), pathological grade (HR 1.963, 95% CI 1.058–3.644, *p* = 0.033) and Masaoka–Koga stage (HR 2.250, 95% CI 1.548–3.272, *p* = 0.000) exerted significant differences in overall survival among 99 patients. In the surgery group, multivariate Cox regression analysis exhibited significant overall survival differences in years at diagnosis (HR 0.563, 95% CI 0.367–0.866, *p* = 0.009), neoadjuvant therapy (HR 0.248, 95% CI 0.071–0.872, *p* = 0.030), radical resection (HR 3.674, 95% CI 1.685–8.008, *p* = 0.001), pathological grade (HR 2.082, 95% CI 1.098–3.947, *p* = 0.025) and Masaoka–Koga stage (HR 2.445, 95% CI 1.607–3.719, *p* = 0.000).

**Conclusions:**

Radical resection and Masaoka–Koga stage were independent prognostic factors for the survival of patients with thymic neuroendocrine tumors. Systemic therapy and integrated management of patients with advanced-stage disease require high-level clinical evidence.

## Background

Neuroendocrine tumors are heterogeneous epithelial proliferative neoplasms with neuroendocrine differentiation. Thymic neuroendocrine tumor (TNET), first proposed by Rosai and Higa [1] in 1972, is a rare disease with strong invasion and relatively poor prognosis compared with other neuroendocrine neoplasms [2, 3] and is often associated with ectopic adrenocorticotropic hormone (ACTH) syndrome (EAS) and multiple endocrine neoplasia (MEN). According to the 2021 World Health Organization classification of thymus tumors [4], TNETs include neuroendocrine tumors (carcinoid/neuroendocrine tumors) (low-middle grade) and neuroendocrine carcinoma (small cell carcinoma and large cell neuroendocrine carcinoma) (high grade). Compared with other tumors, there is a bottleneck in the treatment of TNETs due to the lag in the update of new drugs and clinical treatment experience. The clinicopathological features of TNETs were retrospectively analyzed to reveal real-world outcomes and prognostic factors in a single high-volume clinical center.

## Results

### Patients’ characteristics

A total of 104 patients diagnosed with TNETs during the approximately four decades were eligible with available information. The clinicopathological characteristics of 104 patients with TNETs are shown in Table 1.

**Table 1:** Real-world clinicopathological characteristics of 104 TNET patients

Variables	Value
Gender (male)	66 (63.46%)
Age at diagnosis (years)	45.3 (13.4–74.4)
*Years at diagnosis*	
Before 2000	11 (10.58%)
Between 2000 and 2010	39 (37.50%)
After 2010	54 (51.92%)
Tumor size (centimeters)	5.7 (0.6–30.0)
*Comorbidity*	79 (75.96%)
MEN	14 (13.46%)
EAS	28 (26.92%)
*Surgery*	97 (93.27%)
Radical resection	79 (81.44%)
Palliative resection	18 (18.56%)
Lymph node dissection	47 (48.45%)
Vessel/pericardium dissection	22 (22.68%)
*Pathological grade*	
Low-middle grade	69 (66.35%)
High grade	35 (33.65%)
*Masaoka–Koga stage*	
I	19 (18.27%)
II	13 (12.50%)
III	34 (32.69%)
IV	38 (36.54%)

There were 66 male patients, accounting for 63.46% of all eligible TNET patients. The median age at diagnosis was 45.3 (13.4–74.4) years. The years at diagnosis were divided into three stages: before 2000, between 2000 and 2010, and after 2010, 11 (10.58%), 39 (37.50%), and 54 (51.92%) patients were diagnosed at different year stages, respectively.

The median tumor size was 5.7 (0.6–30.0) centimeters. Smoking history, alcohol consumption history and family history of tumors were retrieved in 34 (32.69%), 15 (14.42%) and 18 (17.31%) patients, respectively. A family history of tumors was more common in patients with MEN, which could be detected by gene sequencing. Fourteen (13.46%) and 28 (26.92%) patients diagnosed with TNETs were confirmed to have MEN (type 1) and EAS, respectively. Other comorbidities, such as primary hypertension, diabetes, and autoimmune diseases, were found in the remaining patients. Dermatomyositis, ataxia syndrome and Lambert-Eaton syndrome were found in several patients diagnosed with TNETs. Nine patients (8.65%) had a simultaneous or heterogeneous second primary cancer, including cervical cancer, liver cancer, teratoma, acinic cell carcinoma of the parotid gland, colon cancer, lung squamous cell carcinoma, breast cancer and cutaneous squamous cell carcinoma (not shown in Table 1).

### Treatment, pathological examination and staging

Ninety-seven (93.27%) patients underwent surgical resection, and the remaining 7 patients did not receive surgery because of unresectable tumors. Video-assisted thoracic surgery was performed in 25 patients. Radical resection was conducted in 79 (81.44%) patients, and lymph node dissection (LND) and vessel/pericardium dissection (VPD) were conducted in 47 (48.45%) and 22 (22.68%) patients, respectively. Of all patients treated by surgery, 11 (11.34%) patients received neoadjuvant therapy, which included chemotherapy in 6 patients, somatostatin analog treatment in 4 patients, and chemoradiotherapy in one patient. Twenty-one (20.19%) cases received puncture biopsy, and 17 (80.95%) of them were consistent with paraffin pathology. Thirty-five (33.65%) patients were examined by intraoperative frozen pathology, while 21 (60.00%) of them were consistent with postoperative pathology.

**Fig. 1:**
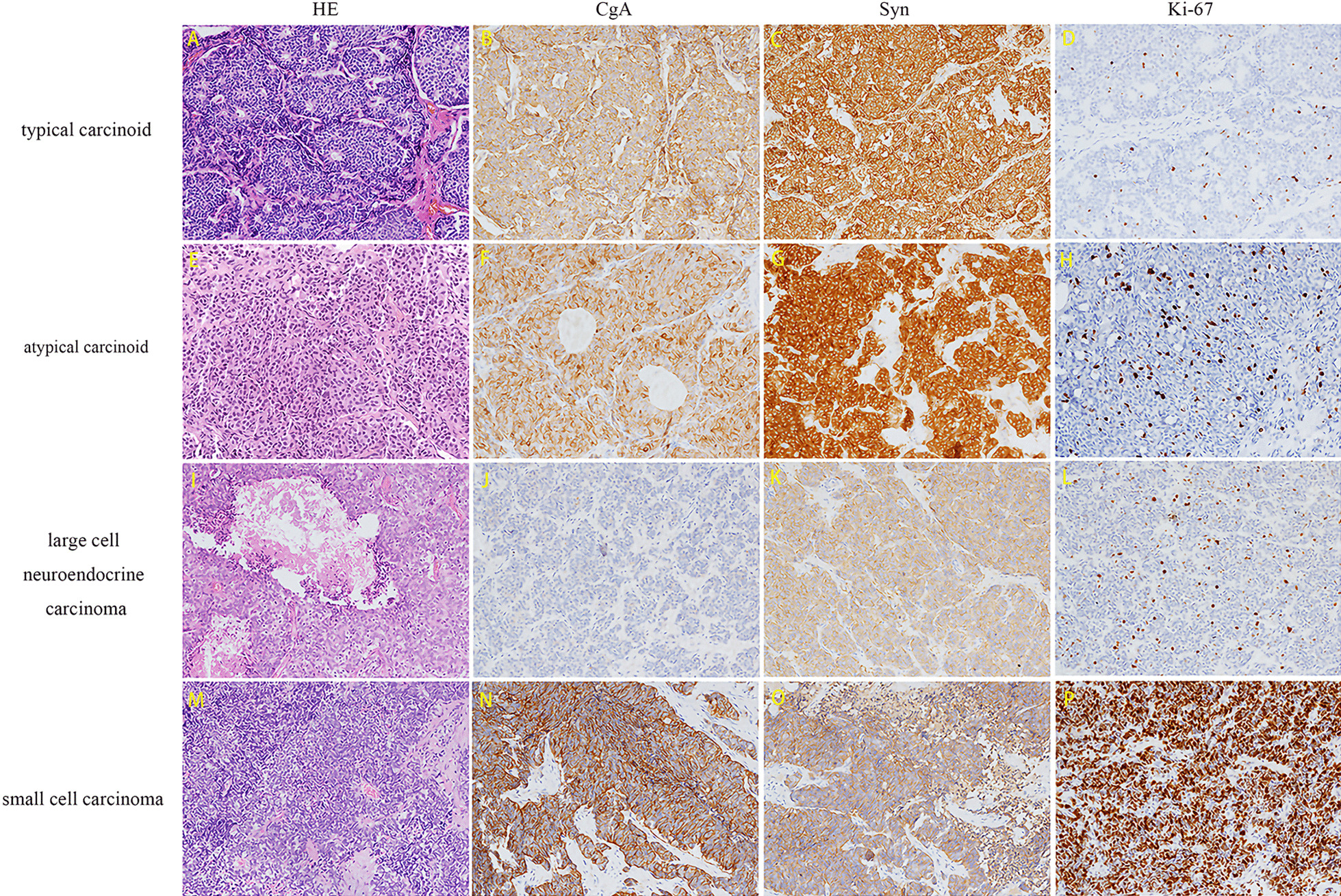
The pathological descriptions of different subtypes of TNETs. **A**, **E**, **I** and **M**, the HE staining of typical carcinoid, atypical carcinoid, large cell neuroendocrine carcinoma and small cell carcinoma, respectively. **B**, **F**, **J** and **N**, immunocytochemistry staining indicated the positive expression of CgA in the four subtypes, respectively. **C**, **G**, **K** and **O**, immunocytochemistry staining indicated the positive expression of Syn in the four subtypes, respectively. **D**, immunocytochemistry staining indicated the 3% of ki-67 expression in typical carcinoid. **H**, immunocytochemistry staining indicated the 5% of ki-67 expression in atypical carcinoid. **L** Immunocytochemistry staining indicated the 5% of ki-67 expression in large cell neuroendocrine carcinoma. **P** Immunocytochemistry staining indicated the 70% of ki-67 expression in small cell carcinoma. All the pictures were shown in 150x. HE, hematoxylin–eosin; CgA, chromogranin A; Syn, synaptophysin

All specimens were confirmed by pathological examination. Sixty-nine (66.35%) patients were classified as low-middle grade TNETs, and 35 (33.65%) patients were regarded as high grade. Mitoses were recorded in 23 patients; the median mitoses were 6 (0–28) per 2 mm^2^. The Ki-67 index was recorded in 65 patients, and the median index was 10% (0%–99%). The pathological descriptions of different subtypes of TNETs were shown in Fig. 1, including hematoxylin–eosin staining of tumor, immunocytochemistry staining of neuroendocrine markers (chromogranin A and synaptophysin) and Ki-67. Lymph node metastasis was found in 35 (74.47% of patients with LND) patients, and involvement of adjacent structures was detected in 61 patients (62.87%). ACTH immunohistochemical staining was performed in 26 patients, of which 18 (69.23%) showed positive expression of ACTH. The corresponding numbers of patients classified into stages I, II, III and IV according to the Masaoka–Koga staging system were 19 (18.27%), 13 (12.50%), 34 (32.69%), and 38 (36.54%), respectively. Patients without surgery were staged according to radiological examination and biopsy pathology.

Adjuvant therapy regimens included chemotherapy, radiotherapy, chemoradiotherapy, somatostatin analog, temozolomide, everolimus, sintilimab, erlotinib and sulfatinib. Some patients were managed in clinical trials.

### Follow-up and survival

**Fig. 2:**
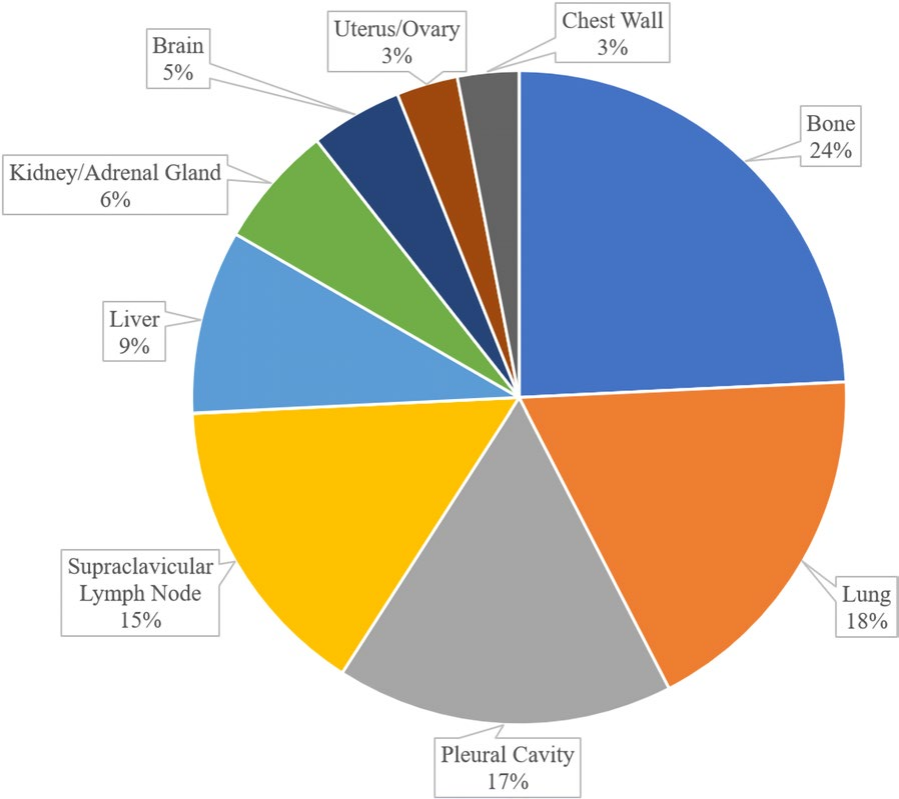
Distribution of metastatic sites in 41 cases during the course of disease

**Fig. 3:**
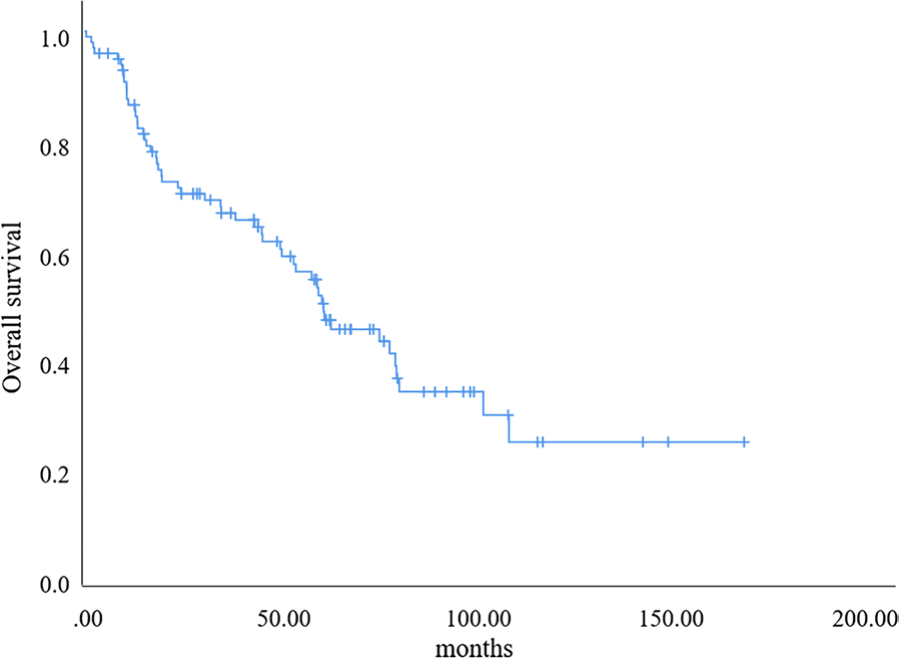
Curve of overall survival analysis of 99 patients with thymic neuroendocrine tumors

The median follow-up time was 69.17 months. By the end of follow-up, 5 patients were lost to follow-up, 53 patients died, and 46 patients survived. Forty-one patients had distant metastases during the course of disease. Different metastatic sites are shown in Fig. 2. The median overall survival (OS) was 61.57 months. The 1-year, 3-year and 5-year OS rates were 91.8%, 70.2% and 54.6%, respectively. The OS curve of 99 patients with TNETs is shown in Fig. 3.

**Fig. 4:**
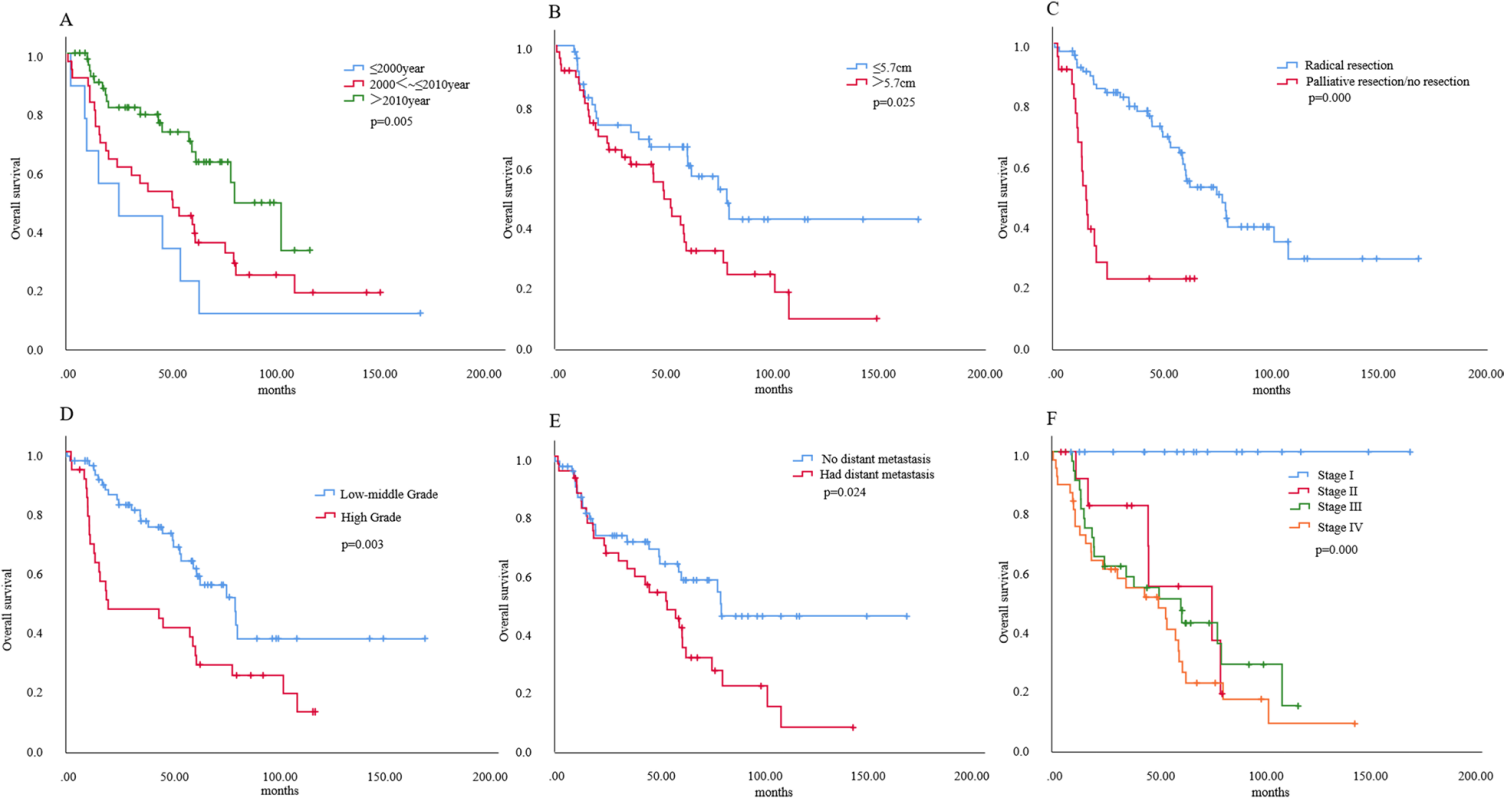
Univariate analysis of overall survival in 99 thymic neuroendocrine tumor patients. **A** Years at diagnosis. **B** Tumor size. **C** Surgical resection. **D** Pathological grade. **E** Distant metastasis. **F** Masaoka–Koga stage

In the univariate analysis of OS among 99 patients with definite follow-up (Fig. 4), years at diagnosis (*p* = 0.005), tumor size (dichotomized around the median) (*p* = 0.025), radical resection (*p* = 0.000), pathological grade (*p* = 0.003), distant metastasis (*p* = 0.024) and Masaoka–Koga stage (*p* = 0.000) showed significant differences in OS. Comorbidity (*p* = 0.030) also showed obvious differences in OS (not shown in Fig. 4).

Upon multivariate analysis (Table 2), years at diagnosis (HR 0.559, 95% CI 0.364–0.857, *p* = 0.008), radical resection (HR 2.860, 95% CI 1.392–5.878, *p* = 0.004), pathological grade (HR 1.963, 95% CI 1.058–3.644, *p* = 0.033) and Masaoka–Koga stage (HR 2.250, 95% CI 1.548–3.272, *p* = 0.000) were identified as independent factors for OS among 99 TNET patients.

**Table 2:** Multivariate Cox regression analysis of 99 TNET patients

Variables	Multivariate Cox regression analysis		
	HR	95% CI	*p* value
years at diagnosis	0.559	0.364–0.857	0.008
surgical resection (radical resection versus palliative/no resection)	2.860	1.392–5.878	0.004
pathological grade	1.963	1.058–3.644	0.033
Masaoka–Koga stage	2.250	1.548–3.272	0.000
Tumor size (≤ 5.7 versus > 5.7)	1.190	0.662–2.141	0.560
comorbidity	0.875	0.427–1.794	0.715

**Fig. 5:**
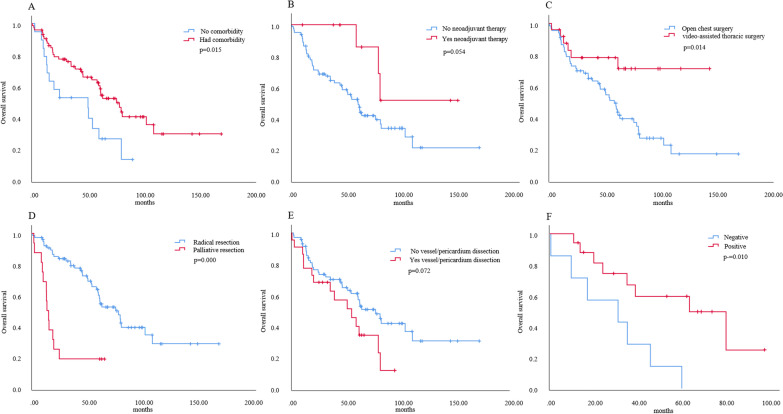
Univariate analysis of overall survival in 92 thymic neuroendocrine tumor patients treated by surgery. **A** Comorbidity. **B** Neoadjuvant therapy. **C** Surgical procedure. **D** Radical resection. **E** Vessel/pericardium dissection. **F** ACTH-Immunohistochemical staining. ACTH, adrenocorticotropic hormone

In the surgery group with 92 patients with definite follow-up, univariate analysis showed that comorbidity (*p* = 0.015), surgical procedure (video-assisted thoracic surgery or not, *p* = 0.014), radical resection (*p* = 0.000), pathological grade (*p* = 0.014) and positive ACTH-immunohistochemistry (*p* = 0.010) exerted significant differences in OS, and neoadjuvant therapy (*p* = 0.054) and VPD (*p* = 0.072) had a potential influence on OS. (Fig. 5). Although lymph node metastasis showed a significant difference (*p* = 0.009) in OS, LND had no significant influence (*p* = 0.204) on OS among patients who underwent surgery (not shown in Fig. 5).

Multivariate Cox regression analysis of 92 TNET patients treated by surgery exhibited significant OS differences in years at diagnosis (HR 0.563, 95% CI 0.367–0.866, *p* = 0.009), neoadjuvant therapy (HR 0.248, 95% CI 0.071–0.872, *p* = 0.030), radical resection (HR 3.674, 95% CI 1.685–8.008, *p* = 0.001), pathological grade (HR 2.082, 95% CI 1.098–3.947, *p* = 0.025) and Masaoka–Koga stage (HR 2.445, 95% CI 1.607–3.719, *p* = 0.000) (Table 3). Regarding the years at diagnosis, Masaoka–Koga stage (*p* = 0.038), radical resection (*p* = 0.000) and tumor size (*p* = 0.053) showed differences among the three different eras.

**Table 3:** Multivariate Cox regression analysis of 92 TNET patients treated by surgery

Variables	Multivariate analysis		
	HR	95% CI	*p* value
years at diagnosis	0.563	0.367–0.866	0.009
Tumor size (≤ 5.7 versus > 5.7)	1.117	0.601–2.077	0.727
comorbidity	0.946	0.448–1.995	0.884
neoadjuvant therapy	0.248	0.071–0.872	0.030
surgical resection (radical resection versus palliative resection)	3.674	1.685–8.008	0.001
vessel/pericardium dissection	1.258	0.632–2.506	0.513
pathological grade	2.082	1.098–3.947	0.025
Masaoka–Koga stage	2.445	1.607–3.719	0.000

**Fig. 6:**
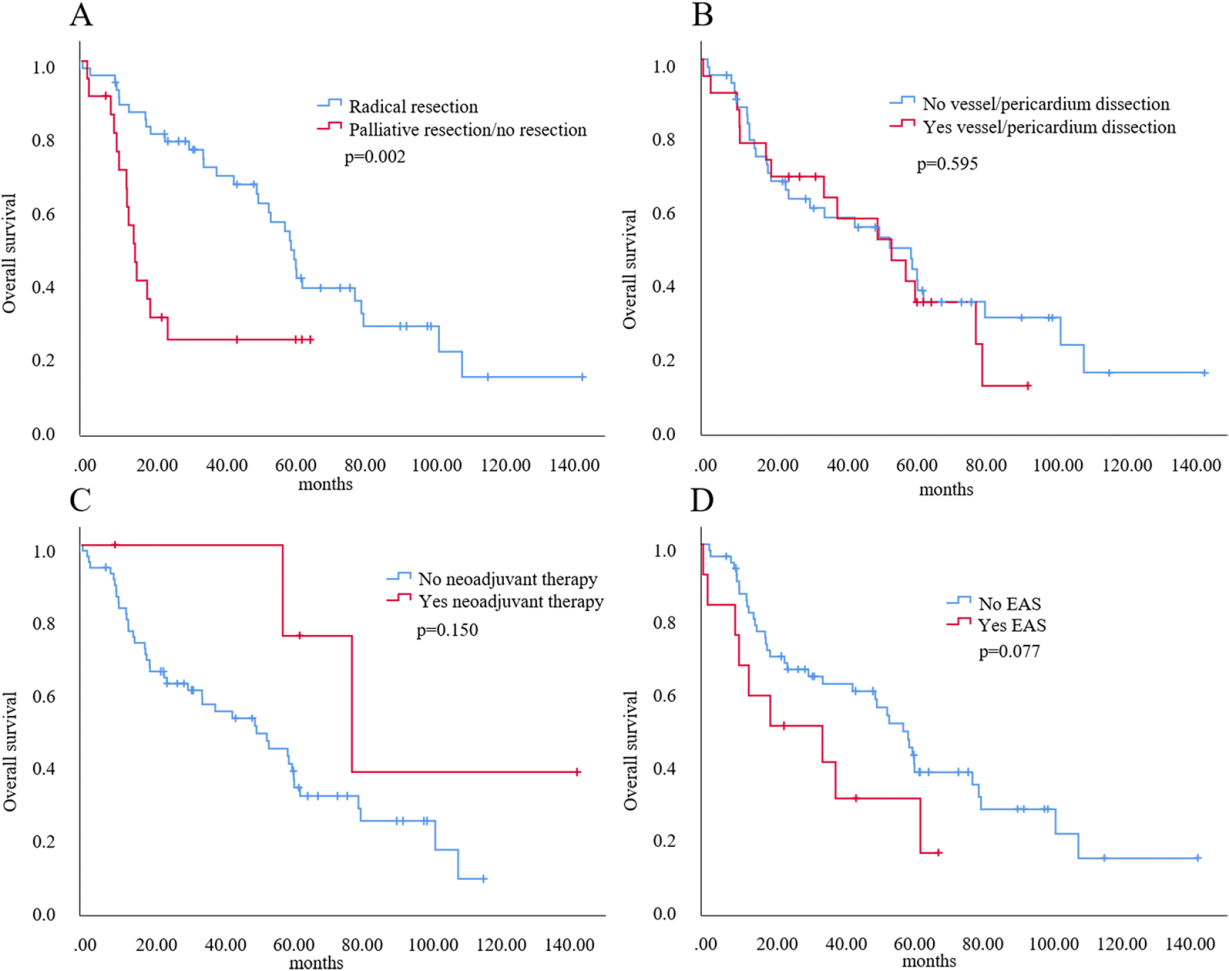
Univariate analysis of overall survival in thymic neuroendocrine tumor patients with Masaoka–Koga stage III/IV disease. **A** Radical resection. **B** Vessel/pericardium dissection. **C** Neoadjuvant therapy. **D** EAS comorbidity. EAS, ectopic adrenocorticotropic hormone syndrome

Patients with Masaoka–Koga stage III/IV could benefit from radical resection (*p* = 0.002). Neoadjuvant therapy (*p* = 0.150) and VPD (*p* = 0.595) did not show significant differences in OS. Patients without EAS seemed to have a potentially superior OS (*p* = 0.077) (Fig. 6).

## Discussion

As a rare setting of diseases, TNETs are usually sporadic, accounting for approximately 0.4% of all NETs and 2–5% of thymic tumors according to different reports [2, 5–7]. TNETs usually present as anterior mediastinal masses with invasiveness. Early-stage tumors with atypical clinical manifestations are often detected by physical examination. Most cases were detected owing to the local invasion of the tumor into adjacent structures.

Histopathological examination is the gold standard for the diagnosis of TNETs. Immunohistochemical staining is essential for accurate diagnosis and pathological typing of TNETs. Given the small biopsy specimens, needle puncture biopsy has some limits on accurate pathological diagnosis [8]. Therefore, the proportion of surgically treated TNET patients in the real world as reported here was relatively high, but the proportion of radical resection was reduced due to the strong invasiveness of tumors. There is a lack of relevant evaluation evidence for the application of intraoperative frozen pathology. While this study indicated that 80.95% (17/21) of patients and 60.00% (21/35) of patients might obtain initial pathological evaluations from puncture biopsy and frozen pathology, respectively, most initial pathologies were neuroendocrine tumors with unclassified subtypes. The specific pathological classification still depended on paraffin pathology. It is difficult to uniformly standardize guidelines for TNETs because of their low incidence and difficulty in diagnosis with small specimens. Most cases in this study obtained pathological confirmation depending on surgical specimens. Biopsy diagnosis was usually confirmed by several pathologists, even via multiple hospital consultations.

Radical resection was performed among 79 (81.44%) patients, and LND and VPD were conducted in eligible patients. Compared with palliative resection or no resection, radical resection (HR 2.860, 95% CI 1.392–5.878, *p* = 0.004) showed a significant difference in OS among 99 TNET patients. A similar result was also seen in TNET patients who underwent surgery and patients with Masaoka–Koga stage III/IV disease. Other previous studies have also indicated the importance of radical resection [9–11]. Due to the uncertain curative effect of systemic therapy and other local therapies for TNETs, complete resection could bring a certain degree of local control, thereby reducing the probability of local recurrence and distant metastasis. However, several questions remain controversary. In this study, LND was conducted in 47 (48.45%) cases, and lymph node metastasis was found in 35 (74.47% of patients with LND) patients, but LND or VPD exerted no significant influence on OS among patients with surgery alone. Several retrospective studies [12–14] comprehensively analyzed populations, including thymic carcinoma and TNETs, suggesting that patients with lymph node involvement had inferior overall survival than patients with negative lymph node metastasis; however, there is a lack of sufficient evidence to demonstrate the positive influence of LND on patient survival. The potential micro metastases which could not be early detected and resected might be a reason for the compromising survival. LND could obtain accurate pathological staging and therefore guide the management of relevant patients. Regarding VPD during surgery for eligible patients, Professor Comacchio and colleagues [15] proposed that the involvement of the great vessels seemed to be associated with a higher recurrence rate without affecting long-term survival. Similar views were also put forward by professor Bertolaccini and colleagues [16] among patients with thymic epithelial tumors (no special TNETs). Although some separate factors, such as LND or VPD, may not bring significant survival benefits for TNET patients alone, they are prerequisites for radical resection. From this retrospective analysis, we suggest that for resectable tumors, radical resection including extending dissection of lymph nodes or adjacent tumor-involved structures should be considered in high-volume surgical centers by experienced thoracic surgeons to provide accurate pathological diagnosis and staging, consequently optimizing the postoperative management of these high-risk TNET patients.

Furthermore, how to improve tumor resectability for advanced stage patients remains unclear. This study indicated that neoadjuvant therapy (HR 0.248, 95% CI 0.071–0.872, *p* = 0.030) had an influence on the OS of TNET patients who underwent surgery, while neoadjuvant therapy was only conducted in 11 patients. In fact, there are no definitive inclusion criteria for patients potentially in need of neoadjuvant therapy; moreover, the lack of evidence-based neoadjuvant therapy regimens for advanced TNETs is also a major problem. Neoadjuvant therapy could be an alternative for unresectable tumors to improve potential resectability under the management of a multidisciplinary team.

Currently, there are no data to provide regimens in the adjuvant setting for TNETs according to different guidelines [17, 18]. Alternative systemic therapy, including platinum-based chemotherapy, somatostatin analogs, temozolomide, capecitabine, etoposide, everolimus, lanreotide and peptide receptor radionuclide therapy with 177Lu-dotatate may be considered in different circumstances for advanced patients. Local adjuvant therapy includes radiotherapy and hepatic regional therapy. Clinical trials are recommended.

This study revealed that years at diagnosis played an important role in the OS of TNET patients. Considering that there were significant differences in Masaoka–Koga stage among different years at diagnosis, chronology showed ordered appropriateness in OS curves, perhaps because more early-stage patients were detected, diagnosed and cared for (more radical resection was conducted) in recent times. Moreover, the limited inspection and treatment methods might also be a factor for inferior survival of TENT patients in earlier years. Thus, early diagnosis and early treatment may be key factors to effectively improve the prognosis of TNETs. Learning from pulmonary nodule screening, it remains unclear whether the popularization of chest computed tomography scanning can decrease overall mortality for other thoracic neoplasms, such as TNETs. We look forward to the therapeutic progress of TNETs.

This study provided the largest single-center study of TNETs to date. However, this study had several limitations. First, it was a retrospective study that lasted for nearly 40 years, which affected the consistency of the patient treatment due to medical levels and limitations in the past and there was a certain degree of bias. Second, due to the rarity and heterogenicity of TNETs, it is difficult to form a uniform treatment strategy for this population. Furthermore, due to the difficulty in diagnosing TNET itself, there were relatively few cases in the non-surgical control group.

## Conclusions

In conclusion, radical resection and Masaoka–Koga stage were important factors in the overall survival of TNET patients. Neoadjuvant/adjuvant therapy and integrated management of diseases require high-level clinical evidence. Clinical trials should be recommended for patients with advanced-stage disease.

## Methods

### Patients

We consecutively evaluated a series of patients who were pathologically diagnosed with TNETs at Peking Union Medical College Hospital from 1983 to 2021. Diagnosis was reviewed and reconfirmed by two pathologists. Patients diagnosed with neuroendocrine tumors of unknown pulmonary or mediastinal origin were eliminated from the study. Patient data, including basic information and schemes for diagnosis and treatment, were retrospectively collected through an electronic medical record system.

### Statistical analysis

Statistical analysis was performed by SPSS software. Measurement data conforming to a normal distribution are presented as‾χ ± s, while nonnormally distributed data are displayed as the median (range). Counting data was exhibited as numbers (percentage). The Kaplan–Meier method was applied to evaluate survival outcome, in which the starting point of OS was set on the date of the diagnosis of TNETs and the ending point was set on the date of death or the date of follow-up. The log-rank test was performed for univariate survival analysis, and the variables with statistical differences in univariate analysis were included in a Cox regression model for multivariate analysis. The difference was considered statistically significant when *p* < 0.05.

## Data Availability

All data generated or analyzed during the study are included in this published article. All the authors retain the raw data and could share them with valid reasons, such as inquiries from readers or academic reports.

## Abbreviations

TNET: Thymic neuroendocrine tumor
ACTH: Adrenocorticotropic hormone
EAS: Ectopic adrenocorticotropic hormone syndrome
MEN: Multiple endocrine neoplasia
LND: Lymph node dissection
VPD: Vessel/pericardium dissection
